# A Structure-Related Fine-Grained Deep Learning System With Diversity Data for Universal Glaucoma Visual Field Grading

**DOI:** 10.3389/fmed.2022.832920

**Published:** 2022-03-17

**Authors:** Xiaoling Huang, Kai Jin, Jiazhu Zhu, Ying Xue, Ke Si, Chun Zhang, Sukun Meng, Wei Gong, Juan Ye

**Affiliations:** ^1^Department of Ophthalmology, The Second Affiliated Hospital, Zhejiang University School of Medicine, Hangzhou, China; ^2^Liangzhu Laboratory, Zhejiang University Medical Center, Hangzhou, China; ^3^NHC and CAMS Key Laboratory of Medical Neurobiology, MOE Frontier Science Center for Brain Science and Brain-Machine Integration, School of Brain Science and Brain Medicine, Zhejiang University, Hangzhou, China; ^4^Alibaba-Zhejiang University Joint Research Center of Future Digital Healthcare, Hangzhou, China; ^5^Department of Ophthalmology, Peking University Third Hospital, Beijing, China; ^6^Beijing Key Laboratory of Restoration of Damaged Ocular Nerve, Peking University Third Hospital, Beijing, China

**Keywords:** glaucoma, visual field, artificial intelligence, deep learning, grading, telemedicine

## Abstract

**Purpose:**

Glaucoma is the main cause of irreversible blindness worldwide. However, the diagnosis and treatment of glaucoma remain difficult because of the lack of an effective glaucoma grading measure. In this study, we aimed to propose an artificial intelligence system to provide adequate assessment of glaucoma patients.

**Methods:**

A total of 16,356 visual fields (VFs) measured by Octopus perimeters and Humphrey Field Analyzer (HFA) were collected, from three hospitals in China and the public Harvard database. We developed a fine-grained grading deep learning system, named FGGDL, to evaluate the VF loss, compared to ophthalmologists. Subsequently, we discuss the relationship between structural and functional damage for the comprehensive evaluation of glaucoma level. In addition, we developed an interactive interface and performed a cross-validation study to test its auxiliary ability. The performance was valued by F1 score, overall accuracy and area under the curve (AUC).

**Results:**

The FGGDL achieved a high accuracy of 85 and 90%, and AUC of 0.93 and 0.90 for HFA and Octopus data, respectively. It was significantly superior (*p* < 0.01) to that of medical students and nearly equal (*p* = 0.614) to that of ophthalmic clinicians. For the cross-validation study, the diagnosis accuracy was almost improved (*p* < 0.05).

**Conclusion:**

We proposed a deep learning system to grade VF of glaucoma with a high detection accuracy, for effective and adequate assessment for glaucoma patients. Besides, with the convenient and credible interface, this system can promote telemedicine and be used as a self-assessment tool for patients with long-duration diseases.

## Introduction

Glaucoma is one of the most frequent causes of irreversible blindness (1, 2), with a high global prevalence reaching 3.54% (3). Early diagnosis and fine grading assessment are of great importance in guiding treatment. Standard automated perimetry is a significant tool for detecting functional damage from glaucoma. The visual field (VF) report obtained from this examination reveals the light sensitivity at different positions in the field of vision and possible optic nerve damage at the corresponding retina regions (4, 5).

Artificial intelligence (AI), particularly deep learning (DL), has been employed in many medical tasks such as medical image analysis and as a clinical decision aid. DL has been often applied in the detection of glaucoma. There have been many reports of the use of AI in the diagnosis via structural changes, including retinal fundus photos (6–11) and optical coherence tomography (OCT) (12–14). Previous studies have focused on function changes as well as VF (15–18) loss. These studies have achieved a high accuracy in glaucoma diagnosis. However, the diversity of the data and complex clinical scenarios remain major hindrances to the promotion of DL in the clinical practice of glaucoma.

Data diversity is the common problem in medical AI, as medical data often come from different hospitals or machines. Previous studies (19–22) have used transfer learning and other DL-based image-reconstruction methods to deal with different types of medical images. For the VF of glaucoma, the frequently used perimeters consist of the Humphrey Field Analyzer (HFA) and Octopus perimeters, which probe the same central 24° or 30° VF with completely different distributions of detection points. Different patterns of input images bring disturbing information to the trained DL models (DLMs), resulting in an often unsatisfactory effect on external validation.

Because clinical situations are complex, a fine-grained grading and comprehensive evaluation are needed to assist in an accurate diagnosis and management. However, clinicians must have a high proficiency in order to interpret fine-grained grading of numerous images in a limited amount of time. On the other hand, it is not difficult for a computer to extract complex features from images and to make decisions rapidly; thus, there is a strong need for DL technology to provide effective grading suggestions for clinicians who are not experienced enough. Previous DL detection (6–10, 12–18) mainly focused on the presence of the disease and showed great results. Yet, more attention should be paid to the precise severity grading of the disease because of its importance in decision making for clinical management. Furthermore, for glaucoma, VF damage reflects only functional impairment, and the relationship between structure and function is important for adequate patient evaluation.

To address the problems outlined above, we developed a fine-grained grading DL system (FGGDL) for glaucoma VF, with a multilevel grading standard. Multiple patterns of data were input into the system from different perimeters, and the VF damage was mapped to the fundus to evaluate patients from two aspects. In addition, we built an interactive interface applied in the FGGDL and valid its auxiliary ability of diagnosis in the real world. This interface can automatically detect progress and provide clinical advice. Our FGGDL may have the potential to provide precise guidance in glaucoma diagnosis and treatment.

## Materials and Methods

This retrospective study is a sub-analysis of VF data from a clinical study (A New Technique for Retinal Disease Treatment, ClinicalTrials.gov identifier: NCT04718532). Ethical approval for the study was obtained from Ethics Committee of ZJU-2 (No Y2020–1027). The research adhered to the tenets of the Declaration of Helsinki and the Health Portability and Accessibility Act.

### Patients and Datasets

We selected 3,805 reliable Octopus VFs (including internal dataset and Real-World dataset) from 2,007 eyes of 1,276 glaucoma patients. Patients underwent VF examination at the Eye Center at the Second Affiliated Hospital of Zhejiang University School of Medicine and the Eye Center of Peking University Third Hospital from February 7, 2013, to August 19, 2020.

The Octopus VFs included in this dataset were measured by two experienced technicians using the G1 program test pattern with stimulus size III by the OCTOPUS 900 perimeter (HAAG-STREIT, Switzerland). The inclusion criteria in this study were as follows: (1) diagnosis of glaucoma by the ophthalmologists, with abnormities in the intraocular pressure (IOP), VFs, retinal fundus photography, OCT, and medical history; (2) without history of glaucoma surgeries; and (3) a false-negative rate ≤ 30% and false-positive rate ≤ 30% for the VFs (23–26). Although patients with severe vision loss typically have a higher false-negative rate (27, 28), the same standard should be implemented to ensure the reliability of other VFs. All VFs were changed to the “right eye” format.

The FGGDL for HFA data (FGG-H) dataset collected a large number of total deviation (TD) values of VF examinations, which contained 13,231 VFs measured by HFA from the Harvard Medical School, which is a publicly available database (29). We excluded VFs that did not concord with the characteristics of nerve fiber bundle abnormalities. We input those data into FGG-H, as described in the “Development of FGGDL and Interface” section.

We collected 150 reliable VFs from the Department of Ophthalmology at the Second Affiliated Hospital of Xi'an Jiaotong University, measured using automated white-on-white perimetry SITA 30-2 fast tests with stimulus size III by HFA (Carl Zeiss Meditec, Dublin, CA). The inclusion criteria were the same as used for the Octopus datasets, with a fixation loss rate ≤ 33% at the same time. To be consistent with the data pattern of the FGGDL dataset, we transferred the 30-2 test pattern into the 24-2 test pattern. These VFs were input into the FGGDL as an external validation dataset.

### Preprocessing of VF Data

Negative values of the comparison graph in the Octopus reports and values of TD in the HFA reports were used in this study, which were converted into Voronoi images using the Voronoi parcellation (30, 31). The value of VF can be arranged in a certain order as vector **x** = [*x*_1_, *x*_2_, ⋯*x*_*k*_], where *k* is the number of test points in the Octopus data or the HFA data. The vector **x**, which is then mapped to the eight-bit grayscale of [0, 255], was converted into vector **y**.

**Figure 1:**
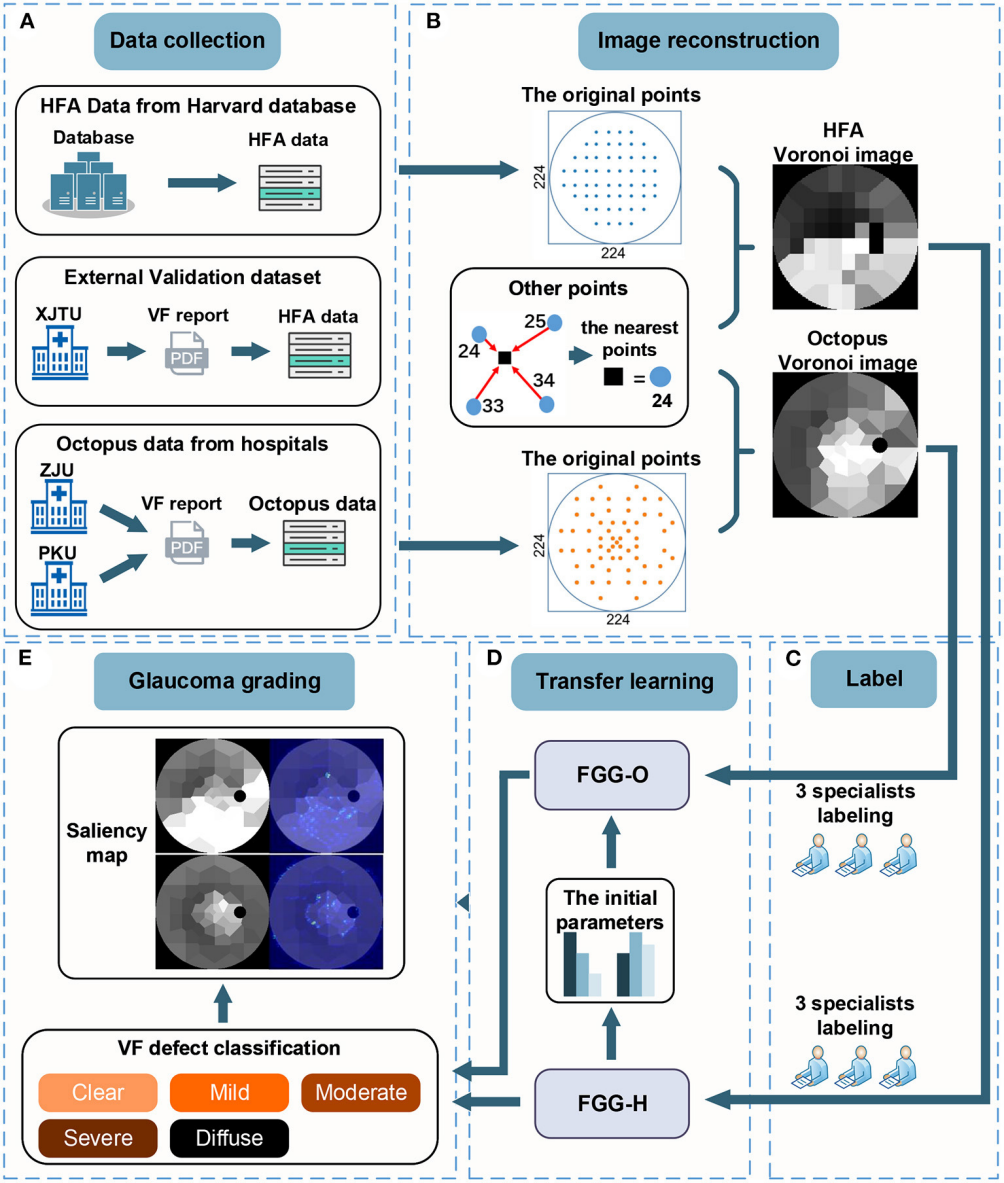
There are five steps in the workflow. **(A)** The data collection step with HFA data from the Harvard database, external validation datasets from XJTU, and Octopus data from ZJU and PKU. **(B)** Process of converting VF data to Voronoi images. **(C)** Process of labeling. **(D)** Transfer learning process, including deep-learning models, FGG-H, and FGG-O. **(E)** Glaucoma grading step consisting of the classification of VF defect and saliency map. VF, visual field; HFA, humphrey field analyzer; VF, visual field; XJTU, xi'an Jiaotong university; ZJU, zhejiang university; PKU, peking university.

To reconstruct the Voronoi images, we built a new 224 × 224 blank image, for which the tangential circle represented the central 30° of VF. The grayscale in the region outside the tangential circle was set to zero. In addition, the points in vector **y** were distributed at their original positions on the VF reports with the grayscale in vector **y**. For other points, its grayscale was equal to the value of the closest points in vector **y**. This process is shown in the “Image reconstruction” (Figure 1).

Moreover, we used data augmentation (32), including flipping the vertical and rotation in the categories with less data, to solve the imbalance of data size over each category, only for the training dataset.

### Classification Standard Reference

The ideal method of VF classification should be objective, reproductive and user-friendly. It should provide effective characteristics of VF defects, including shape, type, location and depth in the same time, have certain grades to directly judge its severity, and be consistent with structure damage. Besides, this method should have enough clinical significance.

Because of the intricate patterns of the VF defect, the standard VF classification is multitudinous. Among the other classifications, the H-P-A scale (33) was acceptable to most ophthalmologists and stipulates that mild glaucoma consists of mean deviation (MD) ≤ −6 dB, moderate glaucoma of −12 dB ≤ MD ≤ −6 dB, and severe glaucoma of MD ≤ −12 dB. Despite its popularity and can be learned in a short time, it still has some disadvantages: the degree of VF defect is roughly and simply divided by MD, which makes the grade could not fully reflect the shape, type and location of VF defect. Besides, the H-P-A standard would be greatly affected when subtle diffuse sensitivity depression exists. The system suggested by the Advanced Glaucoma Intervention Study (23) and Collaborative Initial Glaucoma Treatment Study (34) VF score, two commonly adopted VF scoring system, also have this problem.

In the traditional morphological classification methods of VF, the Aulhorn and Karmeyer five-stage grading method (35) on the basis of a large sample of manual perimetry data is widespread, highly recognized, and still considered to be fundamental reference point in glaucoma research (36). Therefore, based on this method, the nerve fiber bundle abnormalities obtained in the Ocular Hypertension Treatment Study (25) were divided into the five stages as subtypes. This classification method can be applied to Octopus and HFA VF data (Table 1).

**Table 1:** Grading standard of visual field defect of glaucoma.

**Category**	**Name**	**Main characteristic**	**Subtypes**
1	Clear VF	Only relative defects.	
2	Mild VF defect	Spot-like, stroke-like, or arcuate absolute defects, having no connection to the blind spot.	Nasal step defect; Paracentral scotoma defect; Temporal wedge defect
3	Moderate VF defect	Arcuate absolute defects already connected to the blind spot, with or without a nasal break-through into the periphery.	Partial arcuate defect; Arcuate defect; Altitudinal defect
4	Severe VF defect	Extensive ring-shaped or half ring-shaped defects, with a central island of sensitivity maintained.	Double arcuate defect; Tubular vision
5	Diffuse VF defect	Central island collapse, with only the temporal visual field area remaining.	Diffuse defect; Total visual loss

*VF, visual field*.

The ground truth of HFA and Octopus data was generated by three glaucoma specialists according to the proposed standard. Due to the lack of complete VF reports of HFA data of the Harvard database and considering that the model was input with Voronoi data, the use of Voronoi data annotation was conducive to the establishment and validation of the model. On the other hand, Voronoi images enhanced the defect features, makes the annotation of HFA and Octopus data unified as well.

### The Structure Validation of Grading

Glaucoma is relatively complex including functional and structural changes, and it would be easier to determine the overall severity of glaucoma if this grading method could represent the severity of structure to a great extent. To valid the structural consistency of the grading method, we chose 150 cases (30 cases of category 1, 30 of category 2, 30 of category 3, 30 of category 4, 30 of category 5) correctly-classified by FGGDL. These cases had VF reports and retina photos from the same patients, with an examination interval of <3 months. The fundus or VF were divided into 10 sectors or clusters according to the previous research (37). We calculated the number of damaged areas on fundus photos and VF.

### Development of the FGGDL

Due to the reduced amount and imbalance of data of the five categories, we applied transfer learning to solve the loss function misconvergence and overfitting of the network. In view of the high structural similarity between the HFA and Octopus data after Voronoi parcellation, we input HFA data from the Harvard public database to pretrain FGG-H and then constructed another model, the FGGDL for Octopus data (FGG-O), using the same structure. The final parameters of FGG-H were used as the initialization parameters of FGG-O.

Both the FGG-H and FGG-O are classical Resnet-34 (38), which add identity mapping in which the output is composed of the convoluted input and the input itself to avoid the phenomenon of gradient vanishing in this part, as compared with ordinary convolutional neural network (CNN). The input of the entire network passes through a 7 × 7 convolutional layer and a 3 × 3 maximum pooling layer, with both strides 2, realizing the down sampling of the input image. The image then goes through 16 residual blocks, with the number of channels growing from 64 to 512 and the feature map decreasing to a size of 7 × 7. After the average pooling layer, the fully connected layer, and softmax operation, the probabilities of the five categories are output.

There are two types of residual blocks. The input feature map size is *W*× *H*× *C* (width, height, channel). For the first type, the residual mapping part is the convolution of two 3 × 3 convolution kernels, the number of convolution kernels is the same as *C*, and the stride is 1 to remain the size of feature map unchanged. Then the residual mapping part add with the identity mapping part which same with the input, and the output feature map size remains *W*× *H*× *C*. For the second type, the residual mapping part is also the convolution of two 3 × 3 convolutional kernels, but the number of one is 2*C*, the stride is 2, and the feature map would be changed to *W*/2 × *H*/2 × 2*C*. And the number of the other one is 2*C*, the stride is 1. Then the residual mapping part add with the identity mapping part with 1 × 1 convolution kernels, stride of 2, the number of convolution kernels of 2*C*, and the output feature map size is *W*/2 × *H*/2 × 2*C*.

We used cross-entropy as the loss function and added the L2 regularization term to the loss function to reduce the complexity of the network and reduce overfitting. The equation of loss functions is as below:


(1) $$\begin{array}{r} Loss=-\frac{1}{N}\left(\sum_{i=1}^{N}\sum_{c=1}^{M}y_{ic}\log p_{ic}\right)+\frac{\lambda }{2}\sum_{j=1}^{W}\omega_{j}^{2} \end{array}$$

where *N* is the batch size, and each batch is composed of *n* data pairs (*x*_*i*_, *y*_*i*_); *x*_*i*_ represents the input image; *y*_*i*_ (*y*_*i*_∈{1, 2, 3, 4, 5}) is the category of *x*_*i*_, *M* and is the number of categories. The equation of *y*_*ic*_ is as below:


(2) $$y_{ic}=\{\begin{array}{c} 0\;y_{i}\neq c\\ 1\;y_{i}=c \end{array}$$

where *c* represents the category label, *p*_*ic*_ is the probability value that *x*_*i*_ belongs to category *c*, λ is the regularization coefficient of *L*2, and *W* is the number of weights ω that regularization contains.

We trained the FGG-H for 150 epochs with a batch size of 8 and used an initial learning rate of 0.001, adjusted to 0.1 times the original value every 40 epochs, with the regularization coefficient of *L*2 10^−6^. Equally, we trained the FGG-O for 500 epochs with a batch size of 8 and used an initial learning rate of 10^−6^, adjusted to 0.1 times the original value every 50 epochs, with a regularization coefficient of *L*2 10^−4^. We used the Adam optimize gradient descent method in both networks to minimize the loss function. The difference between the two networks was mainly because (1) FGG-O generates the parameters of FGG-H, with a certain feature extraction ability at the beginning, so the small initial learning rate is set to prevent the loss function from oscillating in the training process and (2) the small amount and unbalanced distribution in each category of input data of FGG-O result in a larger regularization coefficient.

### An Interactive Interface and Real-World Performance

To improve the practicability of the FGGDL's assisting clinical decision making, we developed an interface based on the FGGDL and by reading the open-source code of the information on the VF reports (https://github.com/goldengrape/read_medical_device_data) and tested its auxiliary diagnostic capability in the real world.

We performed a diagnostic accuracy study comparing the performance with and without FGGDL assistance. The participating clinicians in the study diagnosed a validation dataset. This dataset including 400 VF reports from 100 patients, and each of them had 4 VF examinations at different times, at intervals of more than 3 months. The clinicians were randomly divided into 2 groups. Group 1 first read the VF report read without FGGDL assistance. And after a washout period of 14 days, they read the VF report with FGGDL assistance in a reverse order. Group 2 first with FGGDL assistance, then without and in the reverse order as well. Clinicians were required to diagnose the grade of VF.

### Statistical Analysis

To compare the diagnostic capability of the FGGDL with that of humans, the same test dataset was provided to them to grade without any clinical materials. The performance of the FGGDL and ophthalmologists was evaluated by three aspects: (1) F1 score (calculated by precision and recall) of each class, (2) the overall accuracy (ACC), and (3) the area under the curve (AUC) of the FGGDL. The performance of the FGGDL-based interface in the real world was evaluated by ACC. We applied a two-tailed paired-sample *t*-test on the ACCs to identify significant differences in performance between the FGGDL and the ophthalmologists. All statistical analyses were performed using SPSS (v26.0, IBM) or Python (v3.6.8, Python Software Foundation). The significance level was designated at 95%, and *p* < 0.05 was considered to be statistically significant. These indexes are defined as follows:

Equations (3)–(6): precision, recall, F1 score, ACC:


(3) $$\begin{array}{rcl} Precision & = & \;\frac{TP}{TP+FN} \end{array}$$

(4) $$\begin{array}{rcl} Recall & = & \;\frac{TP}{TP+FP} \end{array}$$

(5) $$\begin{array}{rcl} F1=\;\frac{2*Precision*Recall}{Precision+Recall} & = & \frac{2*TP}{2*TP+FP+FN} \end{array}$$

(6) $$\begin{array}{rcl} ACC & = & \;\frac{TP+TN}{TP+TN+FP+FN} \end{array}$$

where TP is true positive, TN is true negative, FP is false positive, and FN is false negative.

## Results

### Study Workflow for the FGGDL

A DLM for predicting the degree of glaucoma VF injury must have the following characteristics: (1) handles different types of data for different perimeters, (2) grades the degree of VF injury, and (3) is interpretable by clinicians. Based on the above requirements, we proposed the FGGDL. This system is composed of two DLMs: FGG-H and FGG-O. A suited network was selected for different data patterns.

Figure 1 shows the study workflow. First, we collected and screened out 12,401 HFA VFs from the Harvard public database; 3,405 Octopus VFs from Zhejiang University and Peking University for training, validating, and testing the FGGDL; and 150 HFA VFs from Xi'an Jiaotong University as an external validation dataset (Figure 1A). Second, the two kinds of data were converted into Voronoi images and annotated by three glaucoma specialists (Figures 1B,C). Third, FGG-H was trained and tested based on HFA data and FGG-O based on Octopus data. The final parameters of FGG-H were used as the initial parameters of FGG-O (Figure 1D). Finally, the probabilities of five categories indicating the different degree were output from FGG-H and FGG-O. We used a saliency map for FGG-O to visualize the entire model, indicating the region of interest (ROI) supposed to be located in the area without defect (Figure 1E).

### Demographic Data

There shows an overview of demographic data of the study cohorts. There were 4 datasets in our study: (1) Internal datasets (HFA): This dataset used in the FGG-H consisted of 12401 HFA data in TD values with CSV format from Harvard public dataset, lack of demographic data; (2) Internal datasets (Octopus): 3,405 Octopus VFs included in the FGG-O collected from Zhejiang University and Peking University (Table 2); (3) External dataset (HFA): This dataset included 150 HFA VFs from Xi'an Jiaotong University used as external validation for our FGGDL (Supplementary Table 1); (4) Real world dataset (Octopus): This dataset contained 400 VF reports from 100 patients from Zhejiang University, and each of them had 4 VF examinations at different times, at intervals of more than 3 months (Supplementary Table 2).

**Table 2:** Demographic data of patients in internal dataset of Octopus data.

	**Category**					**Total**
	**1**	**2**	**3**	**4**	**5**	
**Internal dataset - Octopus**						
Patients	185	332	454	483	150	1,221
Age mean (SD)	47.02 (18.57)	48.28 (18.48)	53.19 (18.05)	58.79 (16.10)	58.54 (17.46)	53.72 (18.16)
Male (*n*%)	95 (51.35%)	174 (52.41%)	223 (49.12%)	251 (51.97%)	62 (42.00%)	612 (50.12%)
Eyes	243	434	608	629	189	1,907
VFs	377	677	1,008	1,019	324	3,405
Right (*n*%)	188 (49.87%)	339 (50.07%)	487 (48.31%)	512 (50.25%)	172 (53.09%)	1,698 (49.87%)
MD mean (SD)	−1.26 (0.96)	−3.14 (1.07)	−6.65 (2.19)	−14.07 (4.48)	−16.03 (7.34)	−8.47 (6.33)
sLV mean (SD)	1.96 (0.40)	2.79 (0.74)	5.33 (2.06)	7.06 (1.45)	4.44 (1.95)	4.89 (2.38)

*VF, visual field; MD, mean deviation; sLV, square root of loss variance; right, right eyes; SD, standard deviation*.

### Architecture and Performance of the FGGDL

**Figure 2:**
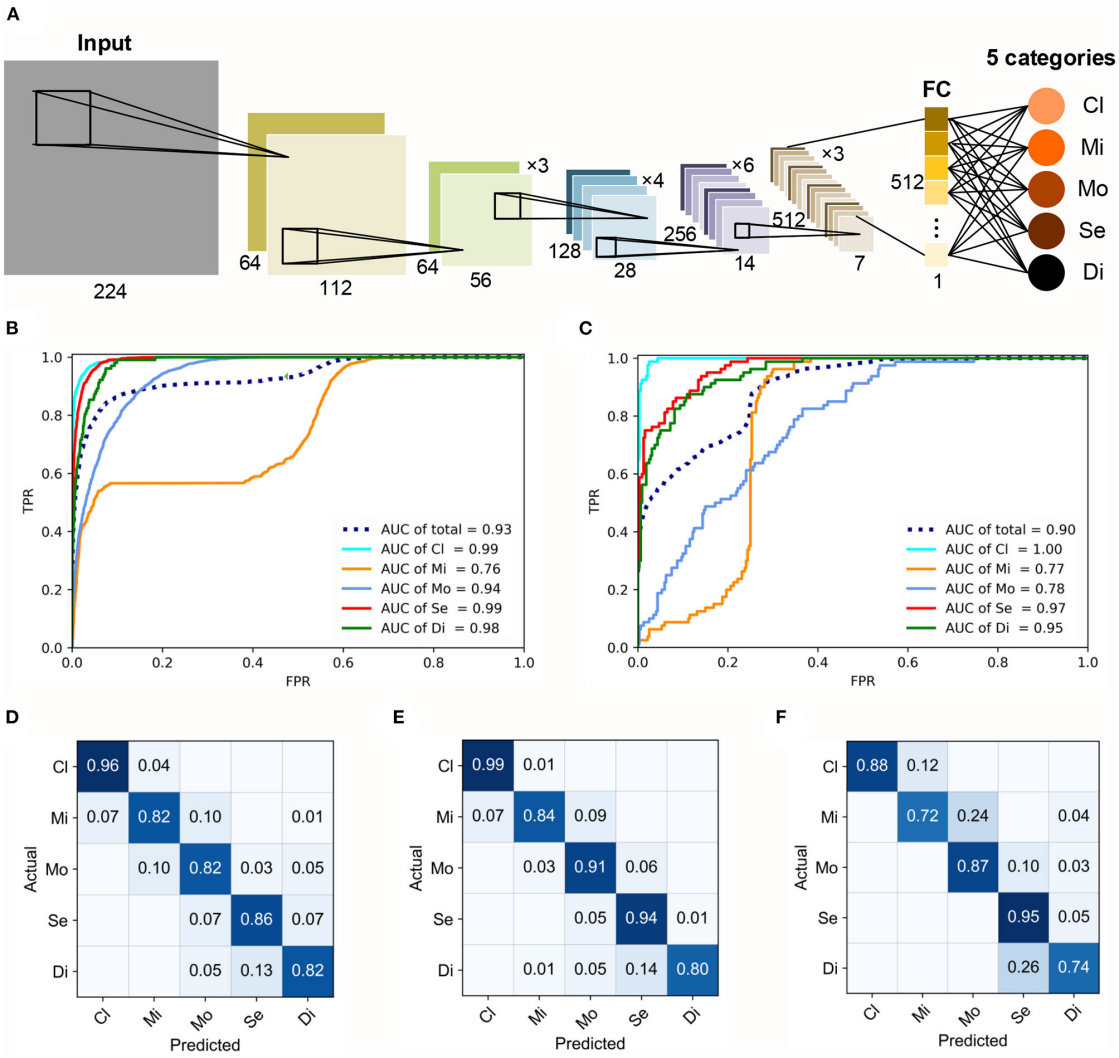
Architecture and performance of the FGGDL. **(A)** Training architecture of the FGGDL framework. The receiver-operating characteristic curves of **(B)** FGG-H and **(C)** FGG-O. The confusion matrixes (CMs) of the predicting results of **(D)** FGG-H, **(E)** FGG-O, and **(F)** external validation datasets. AUC, area under the receiver-operating characteristic curve; Cl, clear visual field (VF); Mi, mild VF defect; Mo, moderate VF defect; Se, severe VF defect; Di, diffuse VF defect.

In our study, the FGGDL showed great capability for both HFA and Octopus datasets. The basic architecture of the FGGDL is shown in Figure 2A. The results of the predicting performance are shown in Figures 2B–F. The FGG-H achieved a total AUC of 0.93 and 0.90 for FGG-O (Figures 2B,C).

For the classification of each level, the ability to identify clear VF, severe VF defect, and diffuse VF defect was shown to be better. For identifying mild defect, both the FGG-H and FGG-O showed weaker performance, with AUCs of 0.76 and 0.77, respectively, probably due to the various subtypes with vastly different shapes included in this grade. The capability of FGG-H for classifying moderate VF defect (AUC = 0.94) was much better than that of FGG-O (AUC = 0.78).

Confusion matrixes (CMs) of FGG-H, FGG-O, and performance on external validation datasets were generated to exhibit the predicting accuracy (Figures 2D–F). For both FGGDL predictions, we found that clear VF could be recognized with the highest accuracy. In general, the total accuracy on the HFA data, Octopus data, and external validation data was 0.86, 0.90, and 0.85, respectively, which shows that the FGGDL could be a good aid in the clinical evaluation of the severity of the VF defect.

### Comparison of the FGGDL With Human Ophthalmologists

**Figure 3:**
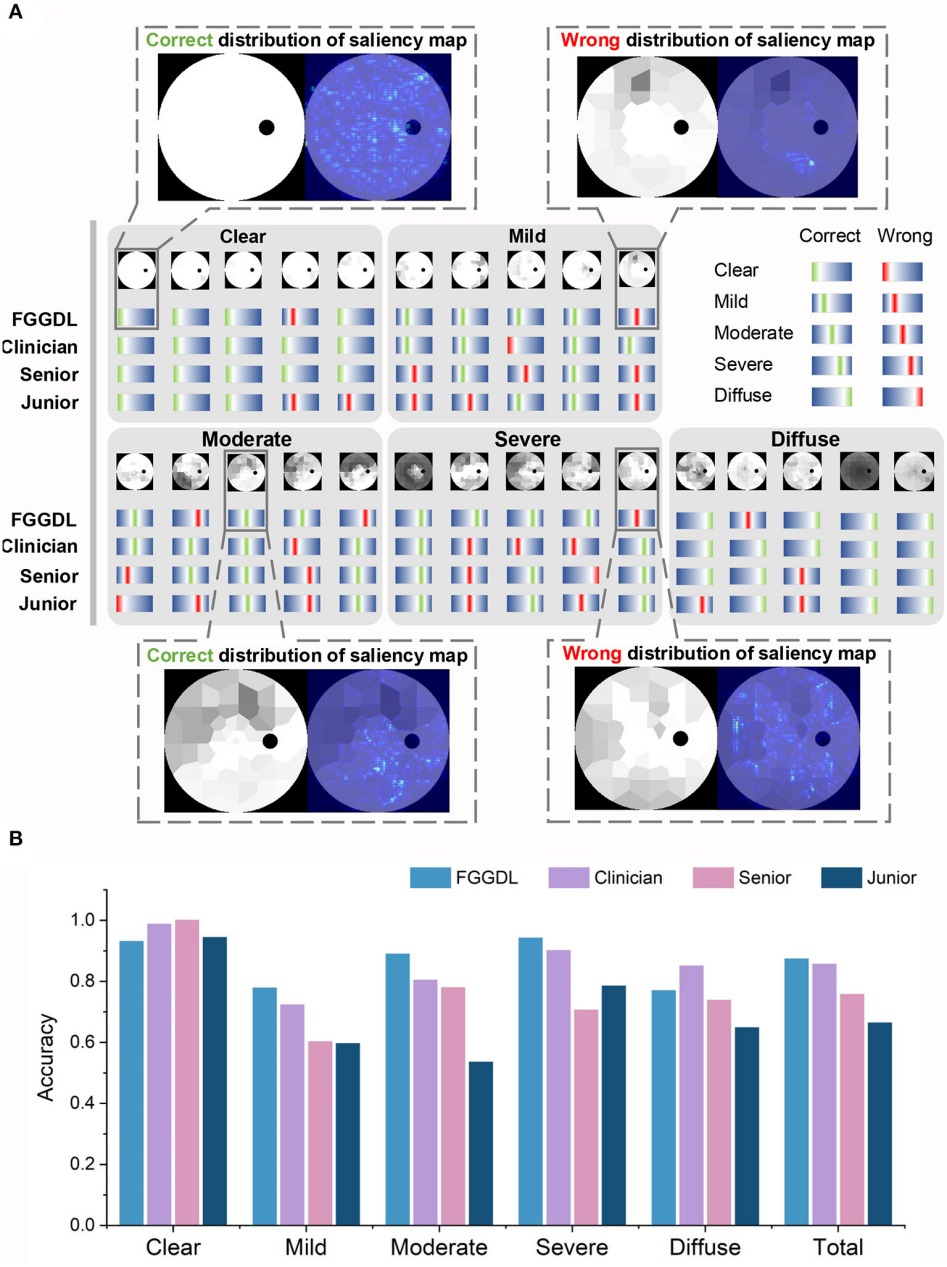
Predicting results of the FGGDL compared with humans. **(A)** Twenty-five selected cases of the predicting results of each category. The red lines represent wrong labels, the green represent correct labels, and the locations of the lines demonstrate the categories. Furthermore, the distributions of the saliency map are shown in the dotted boxes. **(B)** The histogram of the mean predicting accuracy for the Humphrey Field Analyzer data and Octopus data of the FGGDL and three representative ophthalmologists. Senior, senior student; Junior, junior student.

The same test sets, including 400 Octopus datasets and 150 external validation datasets of Humphrey data, were given to seven human ophthalmologists (including three clinicians, two senior medical students, and two junior medical students) to compare the predicting results with those of the FGGDL. It demonstrates examples and statistical results of the diagnostic capability of three representative ophthalmologists (one clinician, one senior medical student, and one junior medical student) and the FGGDL (Figure 3). We selected 25 Octopus cases from the test dataset to exhibit details of the predicting performance. The selected cases covered different subtypes and five cases in each category. Likewise, both incorrect and correct predictions of the FGGDL and human ophthalmologists were covered in these cases (Figure 3A). As compared with the ophthalmologists, the FGGDL showed better predicting performance for the subtype nasal step defect (columns 3 and 5 in Mild), altitudinal defect (column 4 in Moderate), and double arcuate defect (columns 2 and 4 in Severe).

To visualize the focus of our FGGDL, we used the saliency map, and the correct distribution indicated that the ROI was located in the region without defect. For the analysis of the wrong distribution of the saliency map, we found that the shallow diffuse defect caused by cataract and other eye diseases may impede the judgment of the FGGDL. It was easy for FGGDL to treat the cataract defect as a glaucoma VF defect, thus deepening the judgment of the degree of the original defect. In other situations, very shallow tubular defects may be misclassified as other types, possibly because of the unfitting of the profile of the overall deep defect in the category Severe.

It demonstrates the statistical results of the classification accuracy of three representative ophthalmologists and FGGDL (Figure 3B). It shows the mean accuracy of the HFA and Octopus data. The total mean accuracy of the FGGDL was 0.87, which was nearly equal to that of the clinician (0.86) and higher than that of medical students (0.76 and 0.66). Total accuracy and CMs for all human ophthalmologists and FGGDL are shown in detail for HFA and Octopus data, respectively (Table 3; Supplementary Figures 1, 2). We used the F1 score (generally considered the precision and recall) for each category to evaluate predicting performance (Supplementary Tables 3, 4). We found that the FGGDL has the highest F1 score of all categories of the HFA data and for most categories of the Octopus data. It can be proved that FGGDL has excellent classification capability in each category.

**Table 3:** The performance of the FGGDL and humans.

	**Octopus**		**Humphrey**	
	**Accuracy**	***p*-value**	**Accuracy**	***p*-value**
Clinician 1	**0.873** (0.819–0.927)	0.614	0.840 (0.804–0.876)	0.008
Clinician 2	0.800 (0.735–0.865)	0.171	0.800 (0.761–0.839)	0.000
Clinician 3	0.787 (0.720–0.853)	0.114	0.815 (0.777–0.853)	0.000
Senior student 1	0.727 (0.655–0.799)	0.006	0.788 (0.747–0.828)	0.000
Senior student 2	0.700 (0.626–0.774)	0.002	0.775 (0.734–0.816)	0.000
Junior student 1	0.560 (0.480–0.640)	0.000	0.768 (0.726–0.809)	0.000
Junior student 2	0.613 (0.534–0.692)	0.000	0.783 (0.742–0.823)	0.000
FGGDL	0.853 (0.796–0.911)	^*^	**0.895** (0.865–0.925)	^*^

*Accuracy is presented with 95% CIs. Bold text indicates the highest accuracy in each category and overall. HFA, humphrey field analyzer*.

* *There is no p value*.

### Function-Structure Relevance

**Figure 4:**
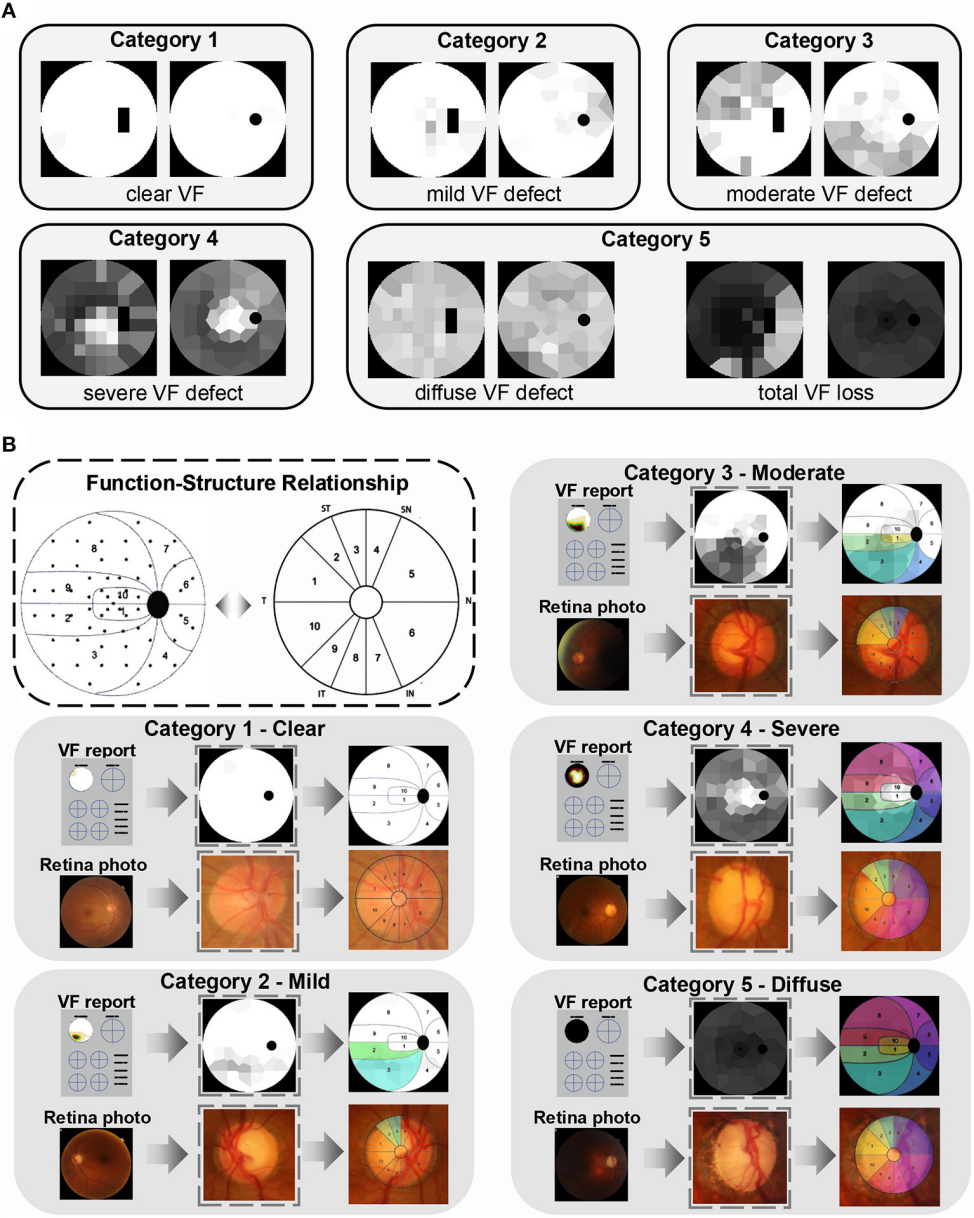
Function–structural relationship of glaucomatous damage. **(A)** Examples of categories 1–5. The left column is the Humphrey Field Analyzer data and the right column is Octopus data. **(B)** The function–structure relationship and the partition in the dotted box were proposed and verified by the previous study. The example of each category is in the gray box. The first row shows the VF reports, converted Voronoi images, and their partition, and the colored area represents the damage. The second row is the corresponding fundus photos, its optic nerve head, and the same colored area demonstrating the related structure damage on fundus.

Different degrees of VF loss in glaucoma are caused by optic nerve damage, which primarily manifest in the optic nerve head (ONH). The FGGDL considered not only the VF injury but also the corresponding fundus injury pattern. It shows examples of the grading standard in the categories Clear, Mild, Moderate, Severe, and Diffuse defect, with transformed HFA Voronoi images in the left column and Octopus in the right (Figure 4A).

Besides, it shows the relationship between VF and ONH damage (Figure 4B) (37). All retina photos of the left eyes were converted into “right eye” format. The same colored area in the VFs and retina photos demonstrated the corresponding defect area. This indicated that the rim loss of ONH increased progressively with augmentation of VF category. This mapping relation between function and structure is strong evidence, which allows for the degree of optic nerve fiber damage to be predicted by the FGGDL at the same time.

We selected 150 cases (30 cases of category 1, 30 of category 2, 30 of category 3, 30 of category 4, 30 of category 5) correctly-classified by FGGDL, and calculated the number of the damaged area on the VF and fundus (Supplementary Table 5). The increased average number of damaged sectors on VF and fundus showed that the degree of structural damage also increased according to this grading method based on VF. In addition, except for category 1 (*p* < 0.01), the numbers of damaged sectors on VFs and fundus united greatly (*p* > 0.01). Perhaps in the early glaucoma, the structural damage precedes functional damage.

### The Performance of FGGDL Application in the Real World

To test the performance of AI assisting clinicians in the real world, we developed an AI assistant interactive interface and did a cross study to valid it (Supplementary Figure 3). It shows the design of this cross-validation study (Supplementary Figure 3A), that clinicians were divided into 2 groups and read the VF reports with (without) AI-assist. After a washout period, they read the same VF reports without (with) AI assistant in a reversed order. And it shows the AI assistant interface based on the FGGDL (Supplementary Figure 3B), which allows clinicians and glaucoma patients to make use of our grading system. This interface can be used to grade the severity of VF, analyze disease progression, and provide clinical follow-up and treatment recommendations. The system then compares it to the previous recorded results according to ID number and eye (left/right), displays whether there is progression, and changes the advice regarding follow-up and treatment. The follow-up and treatment recommendations are based on clinical guidelines (39, 40).

It can be seen from Table 4 that the independent diagnose accuracy of ophthalmologists is lower than that of the FGGDL. However, the accuracy of the senior and junior medical student groups significantly improved when assisted with FGGDL (*p* < 0.01).

**Table 4:** The auxiliary performance of FGGDL interface in the real world.

	**Diagnosis accuracy**		
	**No AI**	**With AI**	***P*-value**
Clinician 1	0.838 (0.801–0.874)	0.885 (0.854–0.916)	0.024
Clinician 2	0.833 (0.796–0.869)	0.875 (0.842–0.908)	0.024
Senior student 1	0.758 (0.715–0.800)	0.798 (0.758–0.837)	0.059
Senior student 2	0.753 (0.710–0.795)	0.863 (0.829–0.896)	0.000
Junior student 1	0.588 (0.539–0.636)	0.820 (0.782–0.858)	0.000
Junior student 2	0.630 (0.582–0.678)	0.758 (0.715–0.800)	0.000
Junior student 3	0.460 (0.411–0.509)	0.813 (0.774–0.851)	0.000
Junior student 4	0.695 (0.650–0.740)	0.875 (0.842–0.908)	0.000
Machine	0.893 (0.862–0.923)		

*Diagnosis accuracy and progression prediction accuracy are presented with 95% CIs*.

## Discussion

Our study has shown the potential for AI-assisted fine clinical grading. Here, we established an effective fine-grained grading system, which we termed FGGDL, to directly guide glaucoma management. This system was verified by multiple data patterns acquired by Humphrey and Octopus perimeters from multiple centers. We also mapped the VF damage to the ONH on fundus, to evaluate the patient in terms of both function and structure and to achieve a diagnosis with much conviction. The FGGDL achieved a high accuracy of 0.85 and 0.90 for the HFA and Octopus data, respectively. We compared the results of the FGGDL with those of human ophthalmologists and found that the FGGDL had significantly superior performance (*p* < 0.01) to that of medical students and nearly equal performance (*p* = 0.614) to the best results of the clinicians. Furthermore, we developed a glaucoma grading AI interactive interface. This system can be used to record the examination report for the same patients to detect the progression of VF and provide corresponding management recommendations applicable in clinical situations. With the assist of this system, the ability of diagnosing had been greatly improved.

The FGGDL combined the VF data from different perimeters of the HFA and Octopus, and the diversity of the data made the results more solid. Transfer learning has recently been widely applied for evaluating different patterns of input data in a specific medical field, such as pneumonia (19), electroencephalogram (41), and cancer genomics (42). In this study, our morphology-based classification method can be applied to both HFA and Octopus data, and we implemented a preprocessing method called Voronoi parcellation and converted the values of TD on VF reports from HFA and Octopus perimeters into similar images with similar features for the CNN. The transfer learning employed afterward ameliorated the network performance on account of the small volume and imbalance of Octopus data, which ensured excellent predicting results of both data types. Thus, the FGGDL was not bound by the difference in data and is inclined to be generalized to the clinical settings.

Effective fine-grained grading for glaucoma VF was applied in the FGGDL to deal with complex clinical situations, including assessment of incipient glaucoma and its subsequent management. Some studies (15–18) have examined the automatic detection of VF defect of glaucoma using different factors, including the values of TD and the probability map of pattern deviation. These studies obtained great performance of their methods, with some being even better than human ophthalmologists. However, because of the lack of a certain grading system, these detection methods were not suitable for obtaining a clinically accurate diagnostic assessment and management. As compared with other methods, the FGGDL judged the severity of VF loss, providing a simple and intuitionistic method for detecting the progression of VF. It further enhanced the auxiliary diagnostic capability of vision loss in glaucoma via the follow-up and treatment decisions provided for clinicians. For instance, for patients with progressed VF, a shorter follow-up time such as 1–2 months is recommended (39). In addition, for patients with advanced glaucoma, surgery with pharmacologic augmentation might be more effective than a generic prostaglandin analog (40).

This AI system connected function and structure to provide a more comprehensive patient evaluation. It can interpret the principles of classification in a more clinical way that is associated with structure damage. The vision loss in VF is considered to be a mapping of the structural damage in the optic nerves of the fundus, appearing particularly in the ONH. Previous studies have detected the structure–function relationship between the corresponding retinal nerve fiber layer and the VF cluster defect. This relationship was presented in the manner of sector area partition in VF and ONH, whether using HFA (43) or Octopus (37, 44), was also applied in the detection of early glaucomatous progression (45), shown in Figure 4B. It can be seen that the number of defective quadrants (inferior, superior, nasal, and temporal) of the ONH increased from category Clear to Diffuse. The change in fundus indicates the progression of optic nerve damage on the fundus, which explains the rationale of the VF defect classification based on the structural changes and simulates the diagnostic thoughts of clinicians.

In our study, we established an AI system based on the FGGDL with a convenient interface. Patients and doctors can upload VF reports and receive the analyzed results, including follow-up and treatment recommendations. In addition, it can detect VF progression, from which the advice would then be changed according to the severity and progression of VF loss. This longitudinal change detection function can better explain the development of the glaucoma course and provide matching clinical advice. In addition, we verified its performance in the real-world, that can significantly improve the ability of ophthalmologists to diagnose and judge progress. Therefore, this AI system can reduce the medical costs of patients, alleviate physician workload, and help achieve goals of telemedicine.

There are several limitations to our study. First, the data size of each category was unbalanced, perhaps because patients with moderate or severe vision loss tend to present to the hospital more frequently. However, we applied data augmentation and transfer learning to solve this problem. Second, the severity of glaucoma could not be defined according only to the functional loss in VFs, and the gold standard should be combined with IOP, structural damage in the fundus via retina photos or OCT, and other clinical information. Hence, the multimodality of the AI diagnosis of glaucoma should be considered in further studies. At last, the high test-retest variability was not considered in our study due to the lack of VF reports tested at the same time of one patient.

In general, we proposed a fine-grained grading AI system based on a novel standard with different data patterns and solid results. This valid, automated multilevel grading achieved potential guidance for the clinical management of glaucoma and was also mapped to structure damage for a more comprehensive assessment of patients. In addition, we developed an interactive interface and validate its practicability in the real world. This will have great potential value in clinical situations such as precision medicine in remote areas. After further research with more clinical validation is applied, this system can promote telemedicine and be used as a self-assessment tool for patients with long-duration diseases.

## Data Availability Statement

Data cannot be shared publicly due to the violation of patient privacy and lack of informed consent for data sharing. Data are available from The Second Affiliated Hospital of Zhejiang University, Department of Ophthalmology (contact JY, yejuan@zju.edu.cn) for researchers who meet the criteria for access to confidential data.

## Ethics Statement

Written informed consent was not obtained from the individual(s), nor the minor(s)' legal guardian/next of kin, for the publication of any potentially identifiable images or data included in this article.

## Author Contributions

XH: methodology, data acquisition and analysis, and writing—original draft. KJ: methodology, data acquisition and analysis, and writing—review and editing. JZ and YX: data acquisition and analysis and algorithm and program design. KS: methodology. CZ and SM: data acquisition and analysis. WG and JY: conceptualization, methodology, project administration, supervision and funding acquisition, and writing—review and editing. All authors have read and approved the final manuscript.

## Funding

This work was financially supported by National Key Research and Development Program of China (2019YFC0118401), National Natural Science Foundation of China (61975178 and 81670888), Zhejiang Provincial Key Research and Development Plan (2019C03020), Natural Science Foundation of Zhejiang Province of China (LR20F050002), Alibaba Cloud, and ZJU-BIOMIND Medical Artificial Intelligence Research.

## Conflict of Interest

The authors declare that the research was conducted in the absence of any commercial or financial relationships that could be construed as a potential conflict of interest.

## Publisher's Note

All claims expressed in this article are solely those of the authors and do not necessarily represent those of their affiliated organizations, or those of the publisher, the editors and the reviewers. Any product that may be evaluated in this article, or claim that may be made by its manufacturer, is not guaranteed or endorsed by the publisher.
