# Supplementary material for: A Structure-Related Fine-Grained Deep Learning System With Diversity Data for Universal Glaucoma Visual Field Grading
Source: Front Med (Lausanne). 2022 Mar 17;9:832920. doi: 10.3389/fmed.2022.832920 (PMC8968343; doi:10.3389/fmed.2022.832920)
Supplement: Supplementary file 1 [file Data_Sheet_1.pdf]

Supplementary Material

1 Supplementary Figures and Tables

1.1 Supplementary Figures

Supplementary Figure 1. Performance of predicting results of humans for Humphrey Field Analyzer (HFA) data. The confusion matrixes (CMs) of predicting accuracy of **(A-C)**, three ophthalmic clinicians, **(D-E)**, two senior medical students, and **(F-G)**, junior medical students. **(H)**, Histogram of the total accuracy of humans compared with the FGGDL for HFA data. Cl, Clear visual field (VF); Mi, Mild VF defect; Mo, Moderate VF defect; Se, Severe VF defect; Di, Diffuse VF defect; Senior, senior medical student; Junior, junior medical student.

Supplementary Figure 2. Performance of predicting results of humans for Octopus data. The confusion matrixes (CMs) of predicting accuracy of **(A-C)**, three clinicians, **(D-E)**, two senior medical students, and **(F-G)**, junior medical students. **(H)**, Histogram of total accuracy of humans compared with the FGGDL for Octopus data. Cl, Clear visual field (VF); Mi, Mild VF defect; Mo, Moderate VF defect; Se, Severe VF defect; Di, Diffuse VF defect; Senior, senior medical student; Junior, junior medical student.

Supplementary Figure 3. (A) The process of the cross-validation study. **(B)** The main operation interface is shown with description of each functional module. The steps 1–3 in the left bottom illustrates the usage of the interface. And the follow-up and treatment recommendations referred to clinical guidelines are shown in the right bottom. VF, visual field; NA, not available; Cl, Clear VF; Mi, Mild VF defect; Mo, Moderate VF defect; Se, Severe VF defect; Di, Diffuse VF defect.

1.2 Supplementary Tables

	category					total
	**1**	**2**	**3**	**4**	**5**	
**External dataset - HFA**						
**patients**	11	23	27	44	22	93
**age Mean (SD)**	37.88 (18.25)	52.52 (20.81)	60.60 (10.11)	61.73 (12.90)	61.74 (16.55)	57.43 (16.79)
**male (n%)**	4 (36.36%)	9 (39.13%)	10 (43.48%)	20 (45.45%)	14 (63.64%)	43 (46.24%)
**eyes**	16	25	30	56	23	150
**VFs**	16	25	30	56	23	150
**right (n%)**	9 (56.25%)	12 (48.00%)	16 (53.33%)	27 (48.21%)	12 (52.17%)	76 (50.67%)
**MD Mean (SD)**	-1.53 (0.91)	-3.03 (0.86)	-6.04 (2.17)	-19.63 (6.90)	-19.88 (11.44)	-12.25 (10.09)
**PSD Mean (SD)**	1.57 (0.18)	2.11 (0.44)	4.28 (2.38)	7.78 (2.15)	3.56 (1.94)	4.82 (3.06)

Supplementary Table 1. Demographic data of patients in external dataset of HFA data. HFA = Humphrey field analyzer; VF = visual field; MD = mean deviation; right = right eyes; SD = standard deviation; PSD = pattern standard deviation.

	category					total
	**1**	**2**	**3**	**4**	**5**	
**Real world dataset - Octopus**						
**patients**	19	30	34	31	17	55
**age Mean (SD)**	44.56 (14.06)	39.54 (13.43)	39.51 (15.79)	49.37 (16.72)	48.32 (16.45)	43.51 (15.85)
**male (n%)**	9 (47.37%)	15 (50.00%)	17 (50.00%)	17 (54.84%)	9 (52.94%)	30 (54.55)
**eyes**	27	45	47	42	19	100
**VFs**	54	103	105	104	34	400
**right (n%)**	29 (53.70%)	45 (43.69%)	57 (54.29%)	55 (52.88%)	14 (48.32%)	200 (50.00%)
**MD Mean (SD)**	0.82 (1.09)	3.00 (1.01)	5.99 (1.68)	13.32 (4.58)	14.52 (7.36)	7.15 (5.98)
**sLV Mean (SD)**	1.87 (0.45)	2.57 (0.60)	4.93 (2.26)	6.92 (1.77)	5.40 (2.32)	4.47 (2.50)

Supplementary Table 2. Demographic data of patients in real world test dataset of Octopus data. VF = visual field; MD = mean deviation; sLV = square root of loss variance; right = right eyes; SD = standard deviation.

	F1 score of each category				
	**1**	**2**	**3**	**4**	**5**
**Clinician 1**	0.929 (0.875~0.981)	0.823 (0.736~0.907)	0.819 (0.736~0.901)	0.788 (0.700~0.876)	0.840 (0.756~0.923)
**Clinician 2**	0.909 (0.845~0.973)	0.686 (0.575~0.796)	0.709 (0.612~0.807)	0.821 (0.758~0.877)	0.873 (0.810~0.930)
**Clinician 3**	0.922 (0.862~0.981)	0.781 (0.685~0.876)	0.733 (0.634~0.831)	0.794 (0.717~0.867)	0.855 (0.776~0.932)
**Senior student 1**	0.847 (0.788~0.900)	0.656 (0.540~0.770)	0.767 (0.685~0.847)	0.785 (0.692~0.874)	0.852 (0.771~0.932)
**Senior student 2**	0.884 (0.832~0.932)	0.667 (0.553~0.779)	0.735 (0.645~0.825)	0.755 (0.656~0.854)	0.811 (0.721~0.900)
**Junior student 1**	0.855 (0.775~0.935)	0.793 (0.705~0.880)	0.729 (0.645~0.810)	0.760 (0.661~0.858)	0.698 (0.589~0.802)
**Junior student 2**	0.873 (0.800~0.946)	0.710 (0.602~0.817)	0.749 (0.668~0.827)	0.788 (0.694~0.879)	0.790 (0.698~0.882)
**FGGDL**	**0.958** (0.916~0.998)	**0.887** (0.817~0.958)	**0.869** (0.796~0.942)	**0.877** (0.808~0.946)	**0.883** (0.814~0.948)

Supplementary Table 3. F1 scores of Humphrey Field Analyzer (HFA) data of FGGDL and humans in each category. F1 scores are presented with 95% CIs. Bold text indicates the highest F1 score in each category and overall.

	F1 score of each category				
	**1**	**2**	**3**	**4**	**5**
**Clinician 1**	0.821 (0.660~0.947)	0.720 (0.531~0.909)	0.830 (0.684~0.969)	**0.973** (0.929~1.016)	**0.894** (0.759~1.028)
**Clinician 2**	0.759 (0.509~1.004)	0.741 (0.565~0.916)	0.772 (0.608~0.935)	0.889 (0.807~0.970)	0.698 (0.487~0.908)
**Clinician 3**	0.788 (0.568~1.008)	0.678 (0.500~0.854)	0.596 (0.383~0.800)	0.893 (0.816~0.967)	0.850 (0.706~0.965)
**Senior student 1**	0.800 (0.633~0.930)	0.630 (0.435~0.824)	0.603 (0.422~0.784)	0.816 (0.708~0.922)	0.750 (0.545~0.949)
**Senior student 2**	0.688 (0.432~0.943)	0.653 (0.450~0.856)	0.625 (0.470~0.776)	0.772 (0.654~0.889)	0.737 (0.525~0.933)
**Junior student 1**	0.681 (0.496~0.825)	0.375 (0.198~0.551)	0.339 (0.157~0.520)	0.739 (0.613~0.858)	0.632 (0.394~0.863)
**Junior student 2**	0.800 (0.594~1.003)	0.776 (0.597~0.953)	0.633 (0.478~0.784)	0.500 (0.338~0.647)	0.492 (0.304~0.678)
**FGGDL**	**0.933** (0.819~1.028)	**0.800** (0.624~0.972)	**0.839** (0.702~0.975)	0.898 (0.821~0.975)	0.756 (0.563~0.948)

Supplementary Table 4. F1 scores of Octopus data of FGGDL and humans in each category. F1 scores are presented with 95% CIs. Bold text indicates the highest F1 score in each category and overall.

category	Damaged sectors		*p* value
	VF	Fundus	
**1**	0.067 (-0.028±0.161)	0.733 (0.330±1.137)	0.003
**2**	1.800 (1.552±2.048)	1.733 (1.342±2.125)	0.702
**3**	3.900 (3.600±4.200)	4.100 (3.703±4.497)	0.206
**4**	7.467 (7.161±7.773)	7.400 (6.955±7.845)	0.745
**5**	9.700 (9.438±9.962)	9.500 (9.227±9.773)	0.161

Supplementary Table 5. The number of damaged sectors is presented with 95% CIs. VF=visual field

2 Other Supplementary Materials

Supplementary Video. The demo video of the interactive interface. It demonstrates the analysis of two visual field (VF) reports of right eye of one patient. And the interface gives the follow-up and treatment recommendation according to the progress.
