# Supplementary figures and images for: A Structure-Related Fine-Grained Deep Learning System With Diversity Data for Universal Glaucoma Visual Field Grading
Source: Front Med (Lausanne). 2022 Mar 17;9:832920. doi: 10.3389/fmed.2022.832920 (PMC8968343; doi:10.3389/fmed.2022.832920)

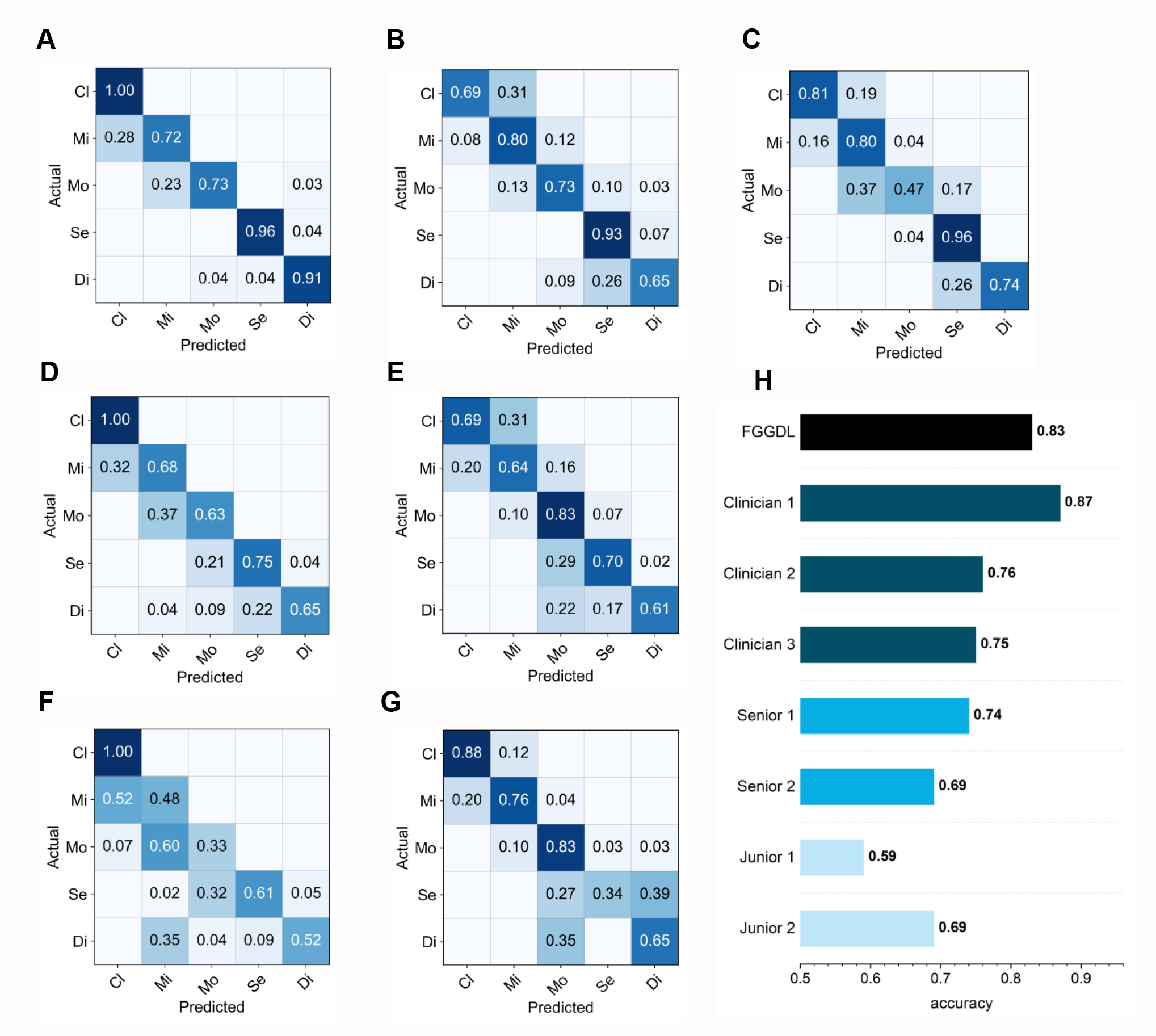

Supplement: Supplementary file 2 [file Image_1.TIF]

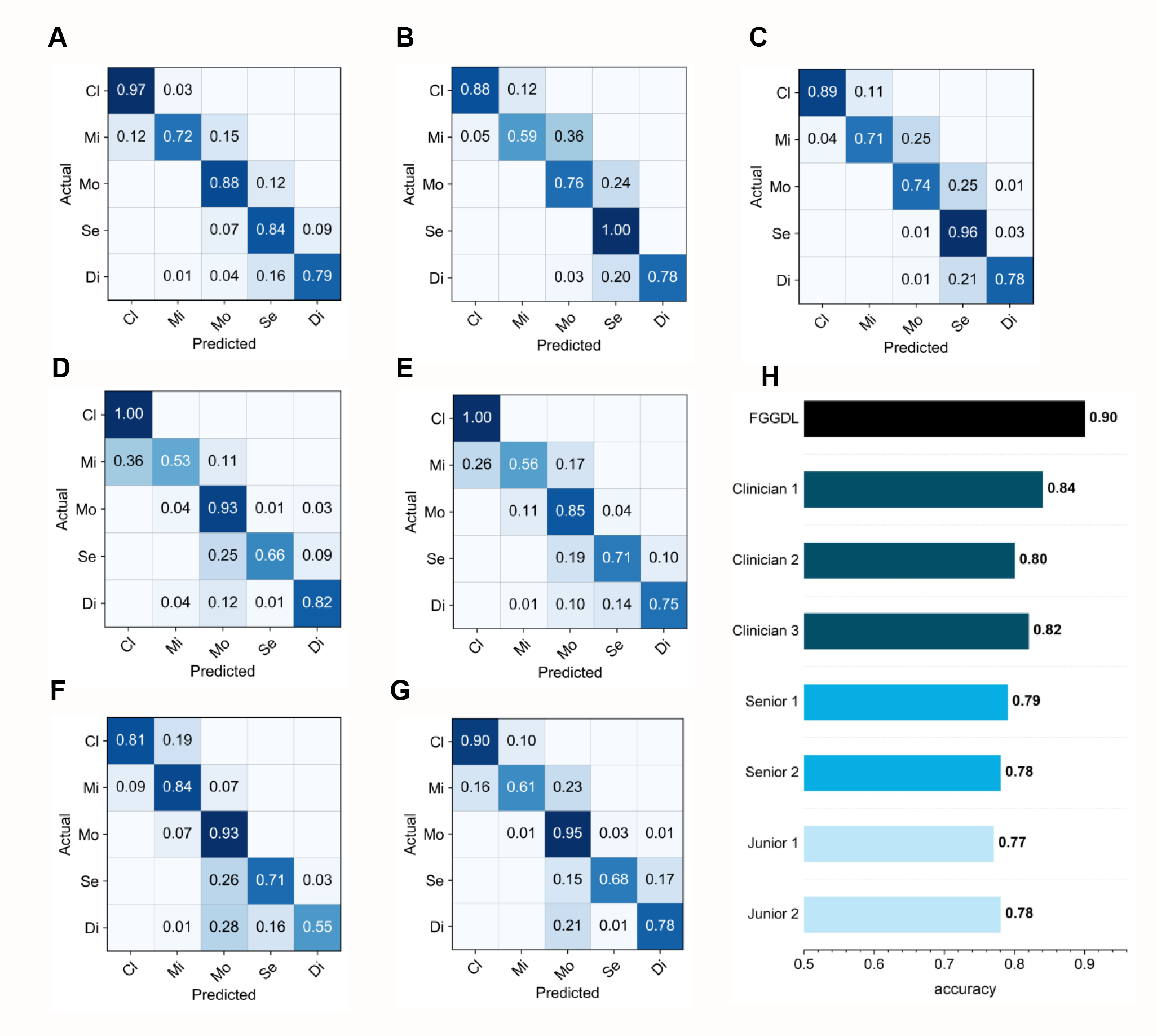

Supplement: Supplementary file 3 [file Image_2.TIF]

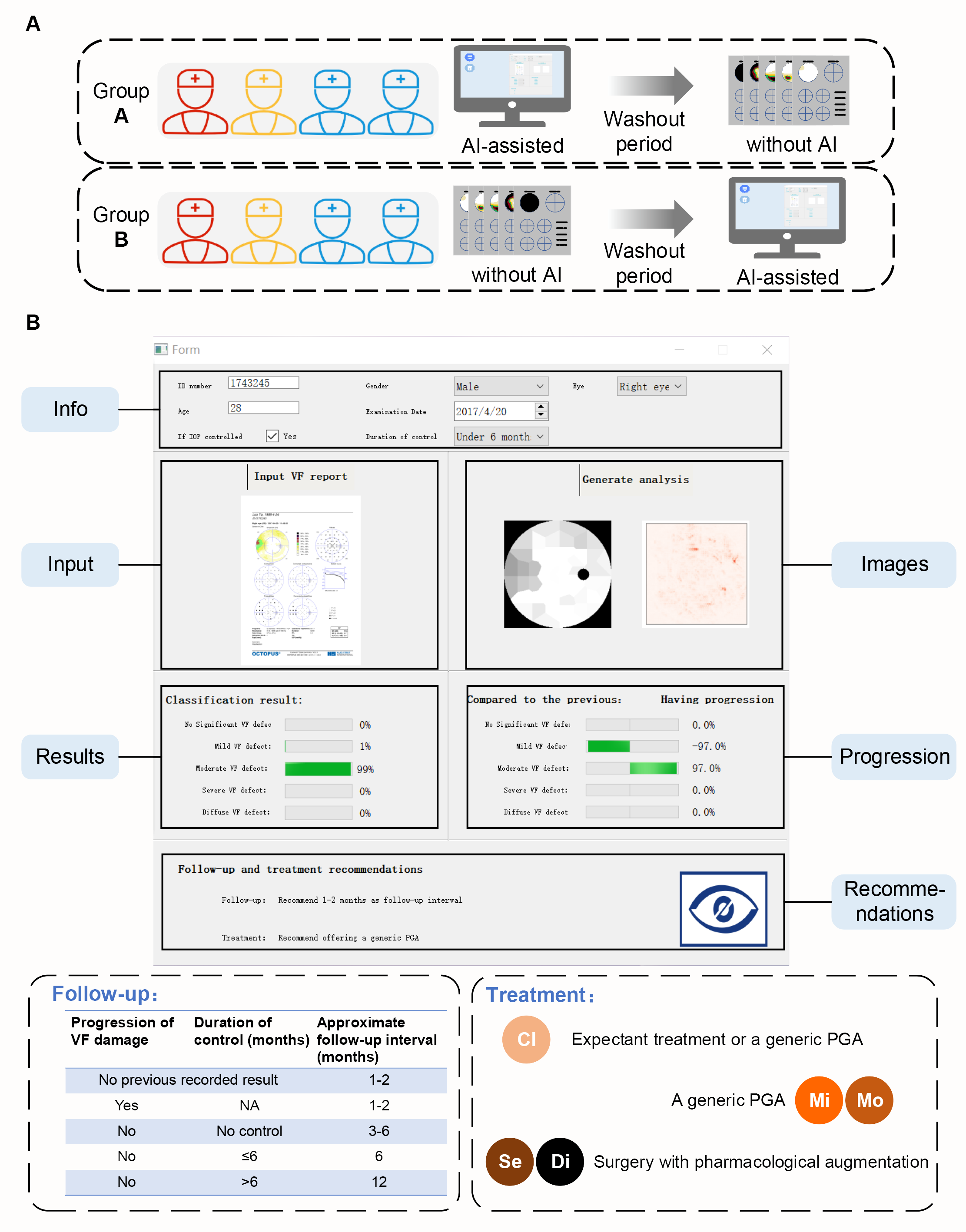

Supplement: Supplementary file 4 [file Image_3.TIF]
